# Endoscopic ultrasound-guided coil and Histoacryl therapy of varices at the hepaticojejunostomy

**DOI:** 10.1055/a-2713-3527

**Published:** 2025-11-05

**Authors:** Thomas Roland Heiduk, André Sasse, Giovanni Federico Torsello, Ali Seif Amir Hosseini, Volker Ellenrieder, Golo Petzold, Ahmad Amanzada

**Affiliations:** 1 27177 Department of Gastroenterology, Gastrointestinal Oncology and Endocrinology, University Medical Center Göttingen, Gottingen, Germany; 2 27177 Department of Clinical and Interventional Radiology, University Medical Center Göttingen, Göttingen, Germany

The development of varices is a common complication of untreated portal hypertension, typically caused by liver cirrhosis or portal vein thrombosis 1 . Uncontrolled portal pressure can result in life-threatening hemorrhages, most often from esophageal or gastric varices 2 . Endoscopic hemostasis or prophylaxis is essential. Treatment options include pharmacologic therapy, endoscopic ligation or glue injection, Sengstaken–Blakemore tamponade, radiological interventions, and surgery 3 . Varices can also occur in other GI segments, such as hepaticojejunostomy (HJS) after pancreatic surgery. Optimal management remains unclear due to the lack of large trials and guidelines 4 .

A patient presented with melena after a prior pylorus-preserving pancreaticoduodenectomy and partial portal vein resection for chronic pancreatitis. Portal vein thrombosis with secondary portal hypertension was evident. External endoscopy showed fresh blood in the small bowel and Grade III esophageal varices without active bleeding. CT imaging confirmed no active hemorrhage but extensive portosystemic collaterals. The patient was referred to the University Medical Center Göttingen.

A transhepatic portal vein stent was placed after successful recanalization, but in-stent thrombosis occurred, and a second attempt failed ( Fig. 1 ).

**Fig. 1:**
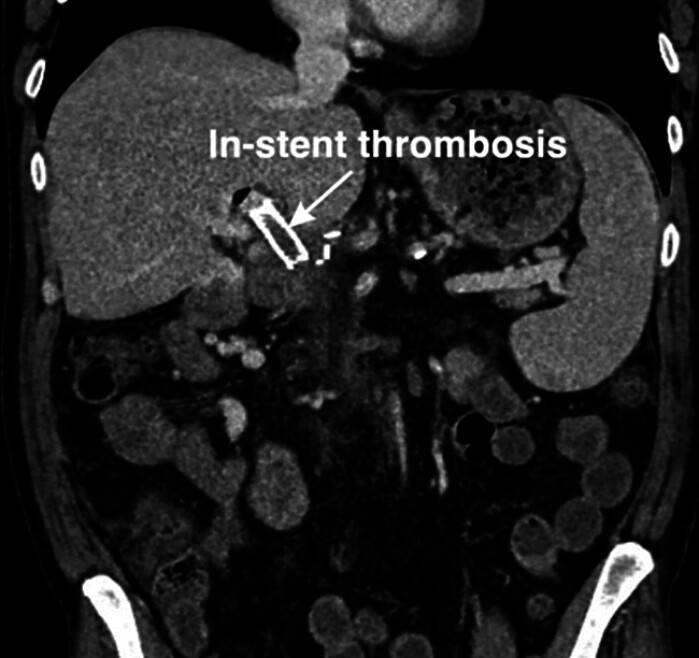
CT imaging confirming In-stent thrombosis.

Endoscopic intervention was then planned. Endoscopy visualized HJS and variceal convolutes. Due to their size, direct puncture was avoided ( Fig. 2 ). A guide wire was placed, and EUS confirmed a large adjacent vessel, supporting EUS-guided intervention.

**Fig. 2:**
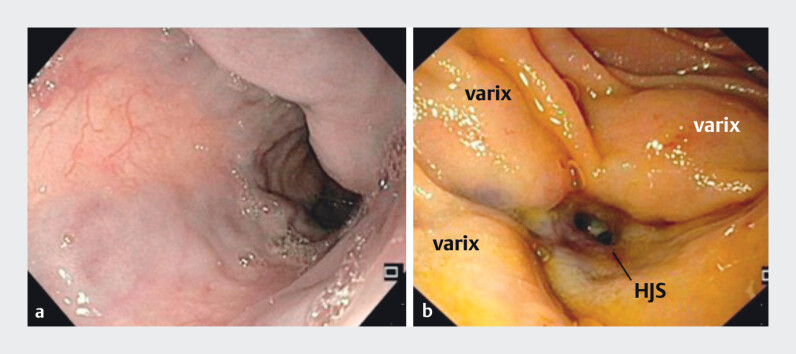
**a** Endoscopic view of esophageal varices and **b** varices at the hepaticojejunostomy site.

One varix was treated with three fibered coils (7 mm each) and Histoacryl; another with two nonfibered coils (8 mm) and Histoacryl ( Fig. 3 ).

**Fig. 3:**
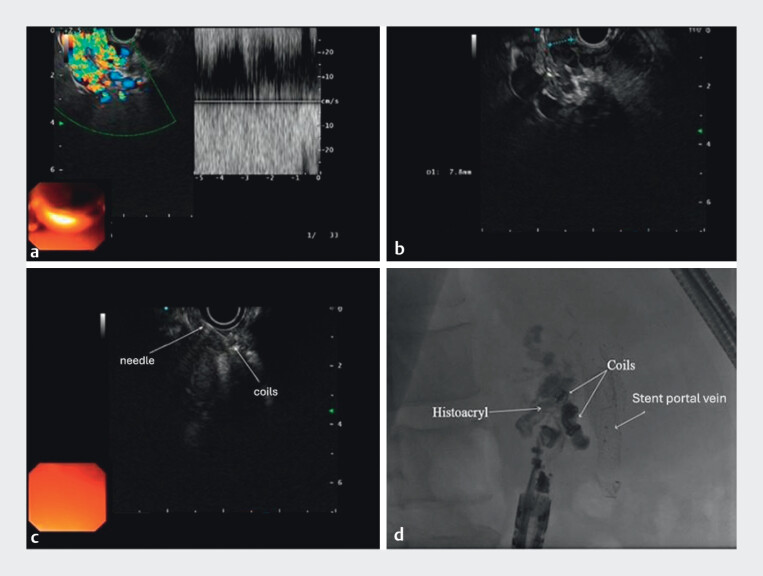
**a** Color Doppler sonographic visualization of hepaticojejunostomy varices; **b** endosonographic measurement of variceal size; **c** puncture of the varix and coil deployment; and **d** radiologic image showing deployed coils and Histoacryl.

Transabdominal ultrasound confirmed success without complications. Follow-up gastroscopy after 3 weeks showed favorable results ( Fig. 4 , Fig. 5 ).

**Fig. 4:**
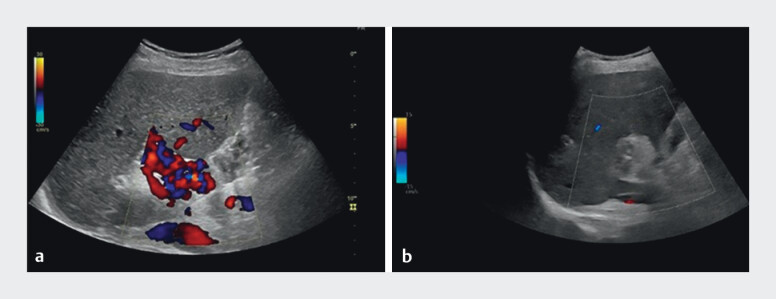
**a** Preinterventional transcutaneous ultrasonography demonstrating varices and **b** postinterventional transcutaneous ultrasonography.

**Fig. 5:**
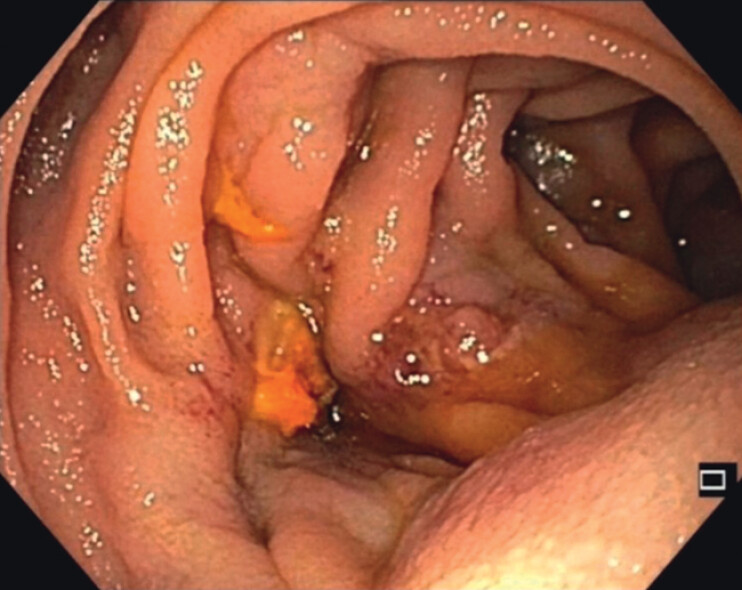
Postinterventional gastroscopic image of the varices at the BDA showing a favorable outcome.

This case demonstrates that endoscopic techniques can effectively treat varices in complex postoperative anatomy ( Video 1 ).

Upper gastrointestinal bleeding control by endoscopic ultrasound-guided coil and Histoacryl therapy of varices at the hepaticojejunostomy in a patient after pylorus-preserving pancreaticoduodenectomy and partial portal vein resection for chronic pancreatitis.Video 1

Endoscopy_UCTN_Code_TTT_1AO_2AD

## Notes

Endoscopy E-Videos https://eref.thieme.de/e-videos
*E-Videos* is an open access online section of the journal *Endoscopy* , reporting on interesting cases and new techniques in gastroenterological endoscopy. All papers include a high-quality video and are published with a Creative Commons CC-BY license. Endoscopy E-Videos qualify for HINARI discounts and waivers and eligibility is automatically checked during the submission process. We grant 100% waivers to articles whose corresponding authors are based in Group A countries and 50% waivers to those who are based in Group B countries as classified by Research4Life (see: https://www.research4life.org/access/eligibility/ ). This section has its own submission website at https://mc.manuscriptcentral.com/e-videos .
